# Whole-genome sequencing in an autism multiplex family

**DOI:** 10.1186/2040-2392-4-8

**Published:** 2013-04-18

**Authors:** Lingling Shi, Xu Zhang, Ryan Golhar, Frederick G Otieno, Mingze He, Cuiping Hou, Cecilia Kim, Brendan Keating, Gholson J Lyon, Kai Wang, Hakon Hakonarson

**Affiliations:** 1 Department of Psychiatry, Zilkha Neurogenetic Institute, Keck School of Medicine, University of Southern California, Los Angeles, CA 90089, USA; 2 School of Bioscience and Bioengineering, South China University of Technology, Guangzhou 510641, China; 3 BGI Tianjin, Tianjin 300308, China; 4 Center for Applied Genomics, Children’s Hospital of Philadelphia, Philadelphia, PA 19104, USA; 5 Stanley Institute for Cognitive Genomics, Cold Spring Harbor Laboratory, New York, NY 11724, USA; 6 Department of Pediatrics, University of Pennsylvania, Philadelphia, PA 19104, USA

## Abstract

**Background:**

Autism spectrum disorders (ASDs) represent a group of childhood neurodevelopmental disorders that affect 1 in 88 children in the US. Previous exome sequencing studies on family trios have implicated a role for rare, *de-novo* mutations in the pathogenesis of autism.

**Methods:**

To examine the utility of whole-genome sequencing to identify inherited disease candidate variants and genes, we sequenced two probands from a large pedigree, including two parents and eight children. We evaluated multiple analytical strategies to identify a prioritized list of candidate genes.

**Results:**

By assuming a recessive model of inheritance, we identified seven candidate genes shared by the two probands. We also evaluated a different analytical strategy that does not require the assumption of disease model, and identified a list of 59 candidate variants that may increase susceptibility to autism. Manual examination of this list identified *ANK3* as the most likely candidate gene. Finally, we identified 33 prioritized non-coding variants such as those near *SMG6* and *COQ5*, based on evolutionary constraint and experimental evidence from ENCODE. Although we were unable to confirm rigorously whether any of these genes indeed contribute to the disease, our analysis provides a prioritized shortlist for further validation studies.

**Conclusions:**

Our study represents one of the first whole-genome sequencing studies in autism leveraging a large family-based pedigree. These results provide for a discussion on the relative merits of finding *de-novo* mutations in sporadic cases *versus* finding inherited mutations in large pedigrees, in the context of neuropsychiatric and neurodevelopmental diseases.

## Background

Autism spectrum disorders (ASDs) are childhood neurodevelopmental disorders characterized by impairments in social interaction, communication, and by restricted, repetitive, and stereotyped patterns of behavior [1]. The Centers for Disease Control and Prevention (CDC) reported in 2012 that approximately 1 per 88 children in the United States has a diagnosis of ASD [2]. Boys are five times more likely to have ASDs than girls. Although autism is typically thought of as a childhood disorder, some affected patients need care even after they reach adulthood. In fact, a recent study demonstrated that it can cost about $3.2 million to take care of an autistic individual over his or her lifetime [3]; therefore, autism presents a great social and economic toll on society.

Understanding the causes of ASDs is critical for the development of better diagnoses and treatment strategies. ASDs are highly heritable and are indeed among the most heritable neurodevelopmental and neuropsychiatric disorders [4]. The genetic basis of ASDs has been pursued aggressively over the past few decades using cytogenetic studies, linkage analysis, and candidate gene association analysis [5]. With the development of high-throughput SNP genotyping technologies, genome-wide association studies (GWAS) [5-9] and copy number variation (CNV) studies [10-13] have been conducted over the past few years, revealing the association between specific candidate genes and loci with ASDs, but with moderate effect sizes.

Recent genetic studies demonstrated that next-generation sequencing (NGS) technology can be a powerful tool to identify the genetic basis of human diseases, especially Mendelian disorders [14-16]. Unlike GWAS that relies on proxy association of genetic variants with unknown disease causal variants, NGS technology enables researchers to interrogate the complete human genome or exome for the detection of both common and rare variants, hence improving the chance of finding disease causal variants, given the potential ability to perform functional annotation on each of the identified variants. Recently, several studies have been published to examine the role of whole-exome sequencing (WES) to identify genetic risk factors for autism. In 2011, a trio-based study of autism performed WES on 60 individuals from families affected with sporadic ASDs and 20 unaffected control individuals, and suggested that *de-novo* sequence variants might contribute to the genetic etiology of ASDs [17]. A follow-up study from the same group sequenced 209 families and found that *de-novo* mutations fall within a highly interconnected β-catenin/chromatin remodeling protein network [18]. A companion paper using WES on 928 individuals, including 200 phenotypically discordant sibling pairs, reported that highly disruptive (nonsense and splice-site) *de-novo* mutations in brain-expressed genes are associated with ASDs and carry large effects [19]. Another study sequenced 175 trios by WES and nominated *CHD8* and *KATNAL2* as genuine autism risk factors, but also suggested a more limited role for the contribution of *de-novo* mutations to ASD pathogenesis than previously reported [20]. Similarly, an exome sequencing study on 343 families did not identify significantly greater numbers of *de-novo* missense mutations in affected *versus* unaffected children, but they identified more gene-disrupting mutations in affected children and found that many of the disrupted genes are associated with the fragile X protein FMRP [21]. The rate of *de-novo* mutations has been recently linked to paternal age, in a study that sequenced 78 trios including 44 offspring with autism and 21 offspring with schizophrenia [22]. Another study sequenced balanced chromosomal translocations in patients with autism or related neurodevelopmental disorders, and revealed the disruption of 33 loci from four categories, reinforcing a polygenic risk model of autism [23]. These and many other recently published studies suggested that *de-novo* mutations may play important roles in susceptibility to autism.

However, current exome sequencing studies on autism may not be comprehensive or representative enough. Many of these studies focus only on simplex families or sequence one affected child from multiplex families. More importantly, the published studies do not specifically analyze inherited mutations, despite the fact that ASDs are highly heritable and that the vast majority of the mutations identified are inherited mutations. We note that one rare exception was published recently, which demonstrated that some familial ASDs were associated with biallelic mutations in known Mendelian disease genes [24]. Although it is clear that *de-novo* mutations explain a fraction of autism patients, it is likely that inherited mutations, in combination or in aggregation, may explain a higher fraction of autism cases. Therefore, we attempted to address this problem by performing a pilot sequencing analysis on patients from multiplex families. We selected a large two-generation family, with parents and eight children, two of whom were diagnosed with autism. DNA samples were available for all subjects, except for one unaffected child. We generated whole-genome sequencing data on the two probands. Not knowing the exact disease model for autism in the family, we performed a series of different procedures for removing variants that are less likely to be functionally important and for finding candidate disease causal genes. Additionally, we genotyped all members of the pedigree (except for the one unaffected child) using Illumina HumanHap550 SNP arrays with approximately 550,000 SNP markers, to help further reduce the number of candidate genes. We have not yet proven whether these mutations singly or in combination contribute to the development of this disease in the two children in this family, and we discuss the potential implications of our study, as a more general issue to the use of NGS for the study of autism and other neuropsychiatric disorders.

## Methods

### Sample selection and sequencing

**Figure 1:**
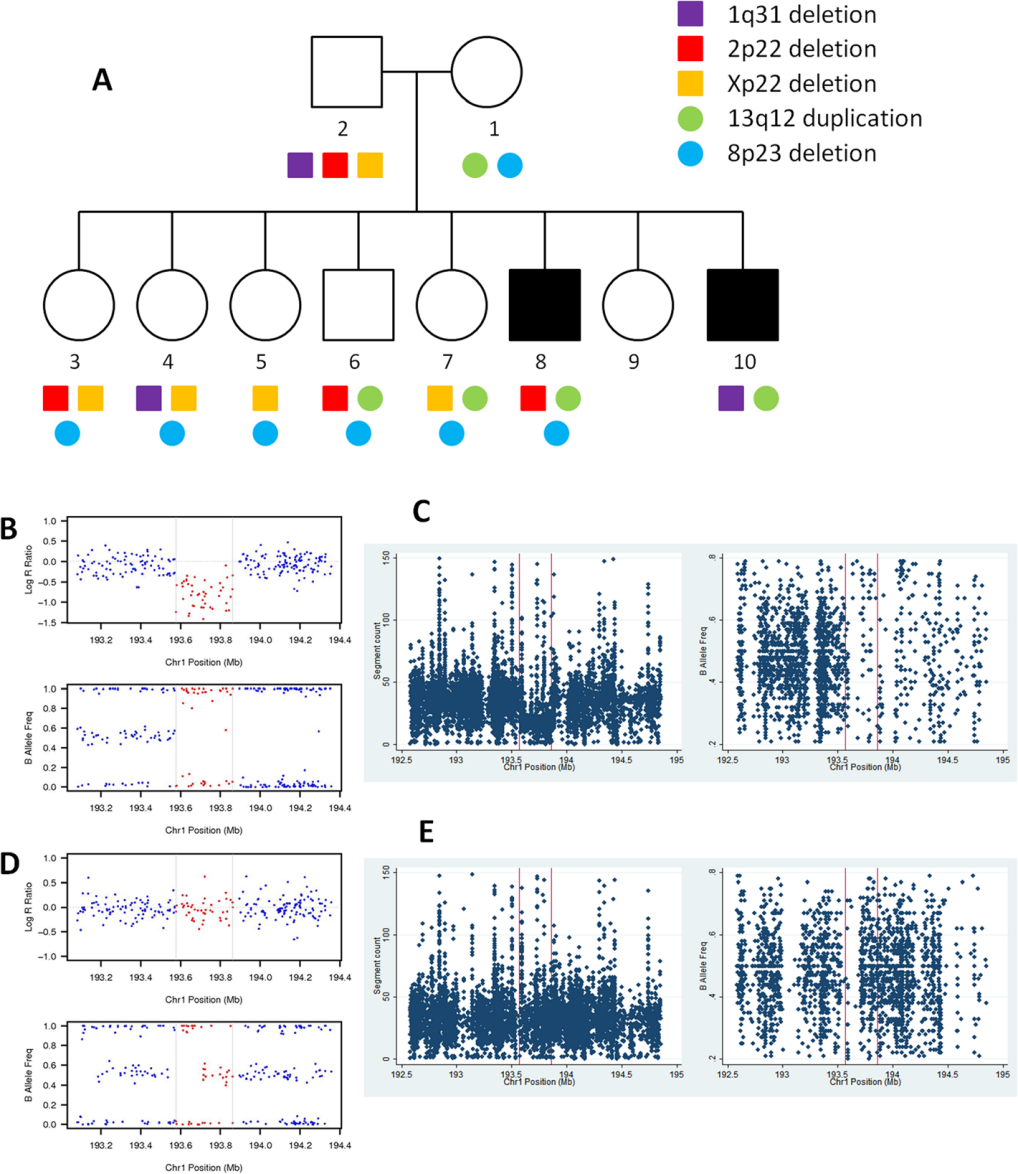
**Analysis of copy number variations (CNVs) in the family with autism.** (**A**) The five inherited CNVs inferred from SNP arrays are depicted with family structure, but none of the CNVs segregate with disease status. (**B**) Signal intensity (Log R Ratio and B Allele Frequency) plot from SNP arrays validates the 1q31 deletion in sample 10. In the deletion (dots between the two vertical lines), Log R Ratio values for SNP markers drop, and B Allele Frequency values cluster around 0 or 1. (**C**) PennCNV-Seq signal (sequence count and B Allele Frequency) plot on WGS data validates the 1q31 deletion in sample 10. In the deletion, the sequence counts tend to be lower than neighboring regions, and very few B Allele Frequency values cluster around 0.5. (**D**) Signal intensity plot from SNP arrays did not indicate the presence of the 1q31 deletion in sample 8. (**E**) PennCNV-Seq signal on WGS data did not indicate the presence of a 1q31 deletion in sample 8.

We manually reviewed all large pedigrees at the Autism Genetic Resource Exchange (AGRE) [25] with >8 subjects, and selected a family for next-generation sequencing. The pedigree includes two parents and eight children, two of whom were affected with autism (Figure 1). The DNA samples for all members of the pedigree were retrieved from the AGRE, and all of them were de-identified subjects. The study was approved by Institutional Review Board of the Children’s Hospital of Philadelphia. After quality control to ensure lack of genomic degradation, we sent 10 ug DNA of two probands to Complete Genomics (CG) in Mountain View, CA, USA for sequencing.

The whole-genome DNA was sequenced with a nanoarray-based short-read sequencing-by-ligation technology [26], including an adaptation of the pairwise end-sequencing strategy [27]. Reads were mapped to the National Center for Biotechnology Information (NCBI) reference genome build 36. The short reads alignment and variant calling were performed by the CG pipeline version 1.7 developed by CG as previously reported [28]. Each variant was assigned a quality score, which was calculated as 10*log_10_[P(call is true)/P(call is false)], representing the confidence in the call. We removed variants that do not pass the default quality filter, including homozygous calls with quality scores <20, or heterozygous calls with quality scores <40. The variants passing the QC threshold were used for downstream analysis.

### SNP genotyping

All genome-wide SNP genotyping for the family was performed using the Illumina HumanHap550 BeadChip at the Center for Applied Genomics at the Children’s Hospital of Philadelphia. Standard data normalization procedures and canonical genotype clustering files provided by Illumina were used to process the genotyping signals and generate genotype calls.

### CNV calling

The Log R Ratio and B Allele Frequency measures for all markers for all samples were directly calculated and exported from the Illumina BeadStudio software. The CNV calls were generated using PennCNV (version 2009Aug27) [29], which utilizes an integrated hidden Markov model (HMM) that incorporates multiple sources of information, including total signal intensity and allelic intensity ratio at each SNP marker, the distance between neighboring SNPs, and the allele frequency of SNPs. Family information was not used in CNV calling. The default program parameters, library files, and the genomic wave adjustment routine [30] in the detect_cnv.pl program were used in generating CNV calls. The scan_region.pl program in PennCNV was used to map called CNVs to specific genes and exons, using the RefSeq gene definitions.

We excluded sparse CNV calls, that is, those CNV calls with average inter-marker distance >50kb (the average distance is approximately 5kb across the whole-genome for the arrays that we used). Furthermore, we excluded all CNV calls whose genomic span overlap with known immunoglobulin regions (chr22:20715572–21595082, chr14:105065301–106352275, chr2:88937989–89411302, chr14:21159897–22090937) was >50%, as these CNVs may be a result of somatic changes. In addition, we excluded CNV calls whose genomic span overlap with centromeres (a list of genomic coordinates for centromeres in human genome NCBI 36 build were given at the PennCNV website FAQ section) was also >50%. The final set of CNV calls encompassing more than or equal to 10 SNP markers were then used in our inheritance analysis.

We also applied a recently published method, ERDS (Estimation by Read Depth with SNVs) version 1.06.04 [31], to generate CNV calls from the sequence data. We first used the Complete Genomics Analysis Tools (http://cgatools.sourceforge.net/) to generate BAM files from CG-provided map files. ERDS starts from read depth information inferred from BAM files, but also integrates other information including paired end mapping and soft-clip signature, to call CNVs sensitively and accurately. Since ERDS models deletions and duplications differently, we collected deletions >10 kb and duplications >200 kb, with a confidence score >300, as a set of highly confident CNV calls. Furthermore, we used a preliminary version of PennCNV-Seq to leverage whole-genome sequence data to validate the CNV calls from SNP arrays. We developed custom scripts to process the BAM file and generated two signal intensity measures: sequence count and B Allele Frequency. Sequence count refers to the normalized sequence read on either a single SNV or as the average across a continuous segment of genomic positions without SNVs, and this measure can be directly counted from SAMtools pileup output. B Allele Frequency refers to the fraction of reads supporting non-reference alleles at a given SNV, and this measure can be calculated from aligned alleles at each position with a SNV call. For 1-copy deletions, one would expect to see decreased sequence count and the general lack of clustering of B Allele Frequency around 0.5, compared to neighboring regions without deletions.

### Validation by Sanger sequencing

Selected putative variants were examined among all family members using Sanger sequencing methods. Given the position of variants, the PCR primers were designed to encompass the candidate position, ensuring that common SNPs are not covered by the primers. The ABI Prism 3500 sequencer was used for sequencing, and the resulting *.AB1 files were loaded into the ABI Sequence Scanner version 1.0 for further analysis and genotype calling. All sequence traces were manually reviewed to ensure the reliability of the genotype calls.

### Variant annotation and prioritization

We used the ANNOVAR software [32] for variant annotation, analysis, and filtering. Besides gene-based annotation, we used a custom ‘variants reduction’ pipeline to identify a list of candidate genes with the following criteria: (1) identify variants causing splicing or protein-coding changes, including stop loss and stop gain variants; (2) remove variants with minor allele frequency (MAF) >1% in the 1000 Genomes Project April 2012 release; (3) remove variants with MAF >1% in the NHLBI-5400 Exomes (European Americans or African Americans); (4) remove variants with MAF >1% in the CG46 database compiled from unrelated individuals sequenced by the Complete Genomics platform; and (5) requiring a recessive mode of inheritance, with at least two deleterious mutations found in each proband.

Additionally, we also used an alternative analytical strategy that attempts to identify any predicted deleterious variants shared by two probands with autism, regardless of disease models or family segregation patterns. We used wANNOVAR [33] (http://wannovar.usc.edu) to process this list of variants, and specified the following criteria in the website: (1) SIFT scores <0.05; (2) PolyPhen scores >0.85; and (3) GERP++ scores >2.0. These are the default thresholds recommended by the developers. The final list of variants and genes are manually examined to identify any prior association with autism or other neurodevelopmental disorders.

To extend the analysis to non-coding variants, we used another custom ‘variants reduction’ pipeline using the ANNOVAR software with the following criteria: (1) identify variants that do not target canonical splicing sites and protein-coding regions; (2) remove variants with minor allele frequency (MAF) >1% in the 1000 Genomes Project April 2012 release, or the NHLBI-5400 Exomes (European Americans or African Americans), or the CG46 database; (3) identify subset of variants that target evolutionarily constrained regions, defined as being located within a GERP++ conserved element with GERP++ scores >2 [34]; and (4) identify subset of variants that target ‘active promoter’ (state 1 inferred by chromHMM [35]) or ‘strong enhancer’ (state 4 and 5 inferred by chromHMM [35]) sites. Given that the nine ENCODE cell lines analyzed by chromHMM do not include a neuronal cell line, we used the data from GM12878 (Epstein-Barr Virus transformed lymphoblastoid cell line), as lymphoblastoid cell lines are used in many gene expression profiling studies on mental disorders.

### Haplotype analysis

We performed haplotype sharing analysis on the pedigree, to identify genomic regions that have an identity-by-descent (IBD) score of 2 between the two affected subjects. Additionally, in an exploratory analysis, we identified regions with IBD score of 0 or 1 between affected and unaffected siblings. We used the Merlin software [36] to perform haplotype phasing on the SNP genotyping data with best estimates of haplotype transmission patterns. We then used a custom script to identify genomic regions that satisfy the user-supplied IBD criteria.

## Results

### CNV analysis on the pedigree

We previously performed whole-genome genotyping on the pedigree, including parents and seven children (DNA samples for subject 9 is not available), using the Illumina HumanHap550 SNP genotyping arrays [6]. Given the availability of signal intensity data from the SNP arrays, we generated copy number variant (CNV) calls (see Methods).

We detected three CNVs in the father and two CNVs in the mother of potential clinical relevance, respectively (Table 1, Figure 1A). Among them, two encompassed genes and both CNVs are inherited from the mother to the offspring. A 50.7 kb duplication on 13q12.13 was detected in the mother and four children. The duplication disrupts the *WASF3* (WAS protein family, member 3) gene, which encodes a member of the Wiskott-Aldrich syndrome protein family. The encoded protein forms a multiprotein complex that links receptor kinases and actin, and is involved in the transmission of signals from tyrosine kinase receptors and small GTPases to the actin cytoskeleton [37]. The *WASF3* gene appears to have the highest expression in brain [38]. A 9.5 kb deletion on 8p23.2 in six children and the mother is located in the intronic region of the *CSMD1* (CUB and Sushi multiple domains 1) gene. A previous report suggests that *CSMD1* may be an important regulator of complement activation and inflammation in the developing central nervous system [39]. The other three transmitted CNVs do not disrupt protein coding regions. However, none of the five CNVs segregate with disease status (Figure 1A), although we cannot exclude the possibility that they may still increase the susceptibility to autism with weak effects. On the other hand, we were not able to identify any *de-novo* CNVs in this family with the array platform that we used, further suggesting that large *de-novo* CNVs are unlikely to be the major cause of autism in this multiplex pedigree. Furthermore, we stress that a *de-novo* CNV is not by any means both necessary and sufficient to cause a disease in any particular individual, as such CNVs have variable expressivity and they are moderated by the genetic background and the environment in each particular family.

**Table 1:** A list of CNV calls encompassing >10 SNPs in the pedigree

**Region (hg18 coordinate)**	**#SNP**	**Length**	**Type**	**ID**	**Start**	**End**	**Relationship**
chr1:193577075-193861997	44	284,923	del	10	rs1359381	rs12745696	Offspring
chr1:193577075-193861997	44	284,923	del	4	rs1359381	rs12745696	Offspring
chr1:193577075-193861997	44	284,923	del	2	rs1359381	rs12745696	Father
chr13:26048387-26099109	10	50,723	dup	7	rs2133814	rs7986966	Offspring
chr13:26048387-26099109	10	50,723	dup	10	rs2133814	rs7986966	Offspring
chr13:26048387-26099109	10	50,723	dup	6	rs2133814	rs7986966	Offspring
chr13:26048387-26099109	10	50,723	dup	8	rs2133814	rs7986966	Offspring
chr13:26048387-26099109	10	50,723	dup	1	rs2133814	rs7986966	Mother
chr2:41082092-41099005	11	16,914	del	6	rs12474136	rs2373974	Offspring
chr2:41082092-41099005	11	16,914	del	8	rs12474136	rs2373974	Offspring
chr2:41082092-41099005	11	16,914	del	3	rs12474136	rs2373974	Offspring
chr2:41082092-41099005	11	16,914	del	2	rs12474136	rs2373974	Father
chr8:3753745-3763223	14	9,479	del	5	rs2930372	rs1464619	Offspring
chr8:3753745-3763223	14	9,479	del	7	rs2930372	rs1464619	Offspring
chr8:3753745-3763223	14	9,479	del	4	rs2930372	rs1464619	Offspring
chr8:3753745-3763223	14	9,479	del	6	rs2930372	rs1464619	Offspring
chr8:3753745-3763223	14	9,479	del	8	rs2930372	rs1464619	Offspring
chr8:3753745-3763223	14	9,479	del	3	rs2930372	rs1464619	Offspring
chr8:3753745-3763223	14	9,479	del	1	rs2930372	rs1464619	Mother
chrX:22775615-22833684	14	58,070	del	5	rs7889437	rs5970944	Offspring
chrX:22775615-22833684	14	58,070	del	7	rs7889437	rs5970944	Offspring
chrX:22775615-22833684	14	58,070	del	4	rs7889437	rs5970944	Offspring
chrX:22775615-22833684	14	58,070	del	3	rs7889437	rs5970944	Offspring
chrX:22775615-22833684	14	58,070	del	2	rs7889437	rs5970944	Father

The DNA sample for subject 9 is not available.

### Whole-genome sequencing identifies a prioritized list of candidate genes

We selected two probands in the family for next-generation whole-genome sequencing by Complete Genomics (CG) with over 50X coverage. In total, we identified 3,811,318 variants (including 3,396,697 SNPs) in proband 1 (ID: 10) and 3,767,904 variants (including 3,365,158 SNPs) in proband 2 (ID: 8), respectively (Additional file 1: Table S1). We next compared these variants to those generated from the Illumina SNP arrays: the concordance rates for proband 1 and 2 were 99.3% and 99.2%, respectively, suggesting the high quality of the sequence data. These high rates of concordance were similar to other published studies using the CG platform [28,40,41].

Given the availability of sequence data, we next explored two methods to generate CNV calls and validate calls from SNP arrays. We first converted the alignment files provided by CG into BAM files, and generated CNV calls using the ERDS software [31]. The CNV calls from SNP arrays can be validated in sequence data, with potentially higher resolution: for example, the boundaries for 1q31.1 deletion (285 kb on SNP array) and 13q12 duplication (51 kb on SNP array) on sample 10 were refined to be chr1: 193574801–193871200 (296kb) and chr13: 26049001–26110000 (61kb), respectively. The list of highly confident CNV calls shared by the two probands is given in Additional file 1: Table S2. Next, we developed a custom pipeline (PennCNV-Seq) to convert BAM files into quantitative measures as ‘sequence count’ and ‘B Allele Frequency’, similar to measures on SNP arrays. The presence (Figure 1B,C) or absence (Figure 1D,E) of CNVs in the two probands can be visually validated by these two quantitative measures, but the data appear to be extremely noisy, highlighting the challenge to generate reliable CNV calls from whole-genome sequencing data.

**Figure 2:**
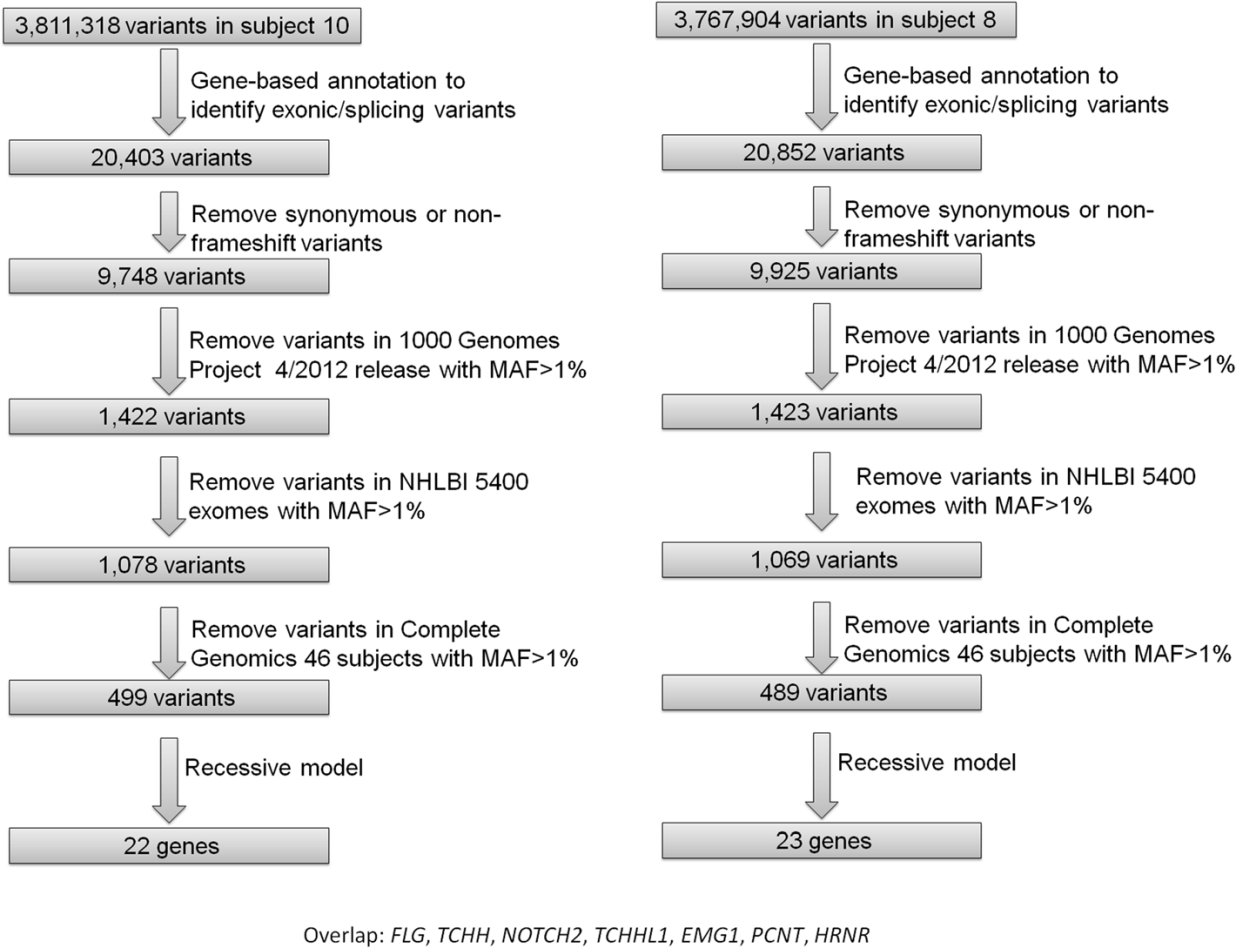
**Illustration of the variants reduction procedure on two probands with autism in the pedigree.** Applying a recessive model of disease inheritance, we identified 22 and 23 candidate genes in the two probands, including seven shared genes.

To identify potentially deleterious mutations from both probands, we next performed variant annotation and prioritization using the ANNOVAR software [32]. Our goal is to identify a list of variants/genes that are likely to be disease causal, and then assess the variant transmission patterns across the pedigree. We used a custom ‘variants reduction’ pipeline on these two genomes, which is composed of a series of procedures (Figure 2). For example, these include removing variants observed in several public databases that compile variant frequency information from large-scale sequencing studies. Similar to a previous study [42], we emphasize here that dbSNP is not used in the filtering procedure, since this database does not contain allele frequency information for the vast majority of SNPs and some disease causal variants may be present in dbSNP. About 500 variants were prioritized to be potentially deleterious using this pipeline in each proband. We next imposed a recessive model, requiring that each gene must contain two deleterious mutations (homozygous or compound heterozygous) to be declared as a putative contributory gene. We implemented a recessive model, because the parents are both unaffected and the phenotype distribution in the siblings is consistent with a recessive model, with 2/8 (25%) of the children being affected. This analysis resulted in 22 and 23 candidate genes in the two children with autism, respectively. Seven of these genes are shared by the two siblings, closely matching the expectation that 25% of genomic region is identical between siblings (Figure 2).

Among these candidate genes, *FLG* (filaggrin) encode an intermediate filament-associated protein that aggregates keratin intermediate filaments in mammalian epidermis [43]. Mutations in this gene are known to cause ichthyosis vulgaris and atopic eczema [44,45]. *TCHH* (trichohyalin) encodes a protein that forms multiple complex cross-links with itself and with other structural proteins, to confer mechanical strength to the hair follicle inner root sheath and to other toughened epithelial tissues [46]. *NOTCH2* (neurogenic locus notch homolog protein 2) encodes a single pass transmembrane protein belonging to an evolutionarily conserved NOTCH receptor family. Mutations in *NOTCH2* have been associated with several developmental diseases. *NOTCH2* mutations are found in about 1% of the cases of Alagille syndrome [47], a severe developmental disorder defined clinically by hepatic bile duct paucity and cholestasis in association with cardiac, skeletal, and ophthalmologic manifestations. Mutations in *NOTCH2* can also cause the Hajdu-Cheney syndrome [48], a disorder of severe and progressive bone loss. Additionally, truncating mutations in the last exon of *NOTCH2* can cause a rare skeletal disorder with osteoporosis [49]. *TCHHL1* (trichohyalin-like 1) has unknown function, but it shared high sequence similarity with *TCHH*. *EMG1* (Essential for Mitotic Growth 1) encodes an essential, conserved eukaryotic protein involved in ribosome biogenesis [50]. Mutations in *EMG1* have been previously associated with Bowen-Conradi syndrome, a lethal autosomal recessive disorder [51]. *PCNT* (pericentrin) encodes a protein that is expressed in the centrosome and is an integral component of the pericentriolar material [52]. Mutations in this gene can cause primordial dwarfism [53]. *HRNR* (hornerin) encodes a component of the epidermal cornified cell envelopes [54], and this gene has been linked with atopic dermatitis (AD) susceptibility in a genome-wide association study [55]. None of these genes are well recognized candidate genes previously associated with autism; however, this is not surprising given some predictions that perhaps approximately 1,000 genes will contribute in some way to the autism spectrum disorders [18-21,24,56].

### Shared haplotype analysis trims down candidate genes

Given the availability of whole-genome SNP genotype data, we next performed haplotype analysis on the pedigree, to assess the utility of using allele sharing information to reduce candidate genes/regions. The goal in this analysis is to identify regions that have IBD (identity-by-descent) =2 in the two affected children, that is, genomic regions that are identically inherited from parents between the two affected children. In theory, only 25% of the genome should have IDB = 2 between the two probands. Using SNP genotype data, we identified 126 genomic regions that fit this criterion, with a total size of 593 Mb. Furthermore, assuming that regions with IBD = 2 have high penetrance for autism and are far less likely to be observed in unaffected siblings, we identified a subset of genomic regions that have IBD = 0 or IBD = 1 between each proband and all other unaffected siblings. This procedure further reduced candidate regions to 27 regions totaling 115 Mb (Table 2). However, we recognize that the latter hypothesis is likely too restrictive, as complex diseases such as autism may behave in polygenic fashion [57,58], that is, true disease causal genes can still be present in IBD = 2 regions in unaffected siblings without manifesting disease phenotypes. This analysis should therefore be regarded as an exploratory analysis to reduce the number of candidate genes to be assessed.

**Table 2:** Genomic regions where the two probands have identical by descent (IBD) of 2, but have IBD of 0 or 1 with all other five unaffected siblings

**Chromosome**	**Start**	**End**	**#SNP**	**Length**	**Start SNP**	**End SNP**
1	24,685,742	25,641,524	174	955,783	rs195704	rs10903129
1	111,171,895	111,330,302	39	158,408	rs343769	rs947633
1	111,345,660	118,691,338	1,454	7,345,679	rs12038954	rs7535961
1	118,704,719	143,649,677	333	24,944,959	rs10923556	rs2500347
1	144,148,243	144,975,558	40	827,316	rs2236566	rs12122100
1	156,202,557	165,715,016	2,338	9,512,460	rs16839492	rs7518703
1	201,467,879	204,068,495	606	2,600,617	rs6672661	rs1361754
1	204,074,127	214,016,229	2,022	9,942,103	rs954206	rs7549052
2	143,712,980	143,772,718	16	59,739	rs4371294	rs12328672
3	71,274,040	71,332,365	26	58,326	rs4677532	rs7374975
3	188,567,203	189,810,377	350	1,243,175	rs6797770	rs3732909
3	189,812,552	191,786,499	536	1,973,948	rs9824282	rs6444435
5	179,518,398	179,998,061	106	479,664	rs6897922	rs4700745
5	180,003,882	180,623,543	95	619,662	rs11960332	rs1279912
8	13,440,994	15,451,587	1,070	2,010,594	rs1160220	rs919401
8	15,464,497	17,859,195	885	2,394,699	rs12547525	rs208753
8	17,881,369	18,740,036	391	858,668	rs10503606	rs6982585
10	53,836,193	58,170,063	976	4,333,871	rs11001909	rs10825864
10	58,698,423	63,455,095	941	4,756,673	rs2393230	rs10821944
10	109,396,522	114,694,771	1,012	5,298,250	rs11193576	rs17746916
12	14,293,625	17,541,979	550	3,248,355	rs17834211	rs1553115
12	17,545,101	28,831,294	2,813	11,286,194	rs10840729	rs7311230
12	130,049,943	132,288,869	432	2,238,927	rs7135850	rs7975069
13	59,004,926	61,882,091	525	2,877,166	rs1622710	rs11838572
13	62,305,059	66,409,431	620	4,104,373	rs9598515	rs9540948
13	66,611,634	73,911,046	1,636	7,299,413	rs7336017	rs9573384
13	73,937,508	77,639,621	839	3,702,114	rs9318278	rs2254690

With the above analysis, we found that *NOTCH2* (neurogenic locus notch homolog protein 2) is the only gene among the seven candidates that fall within the 27 candidate regions. Sanger sequencing confirmed that the two probands share a R2047W mutation in exon 34 and D1327G in exon 24 of *NOTCH2*. However, while R2047W is present in the father, both parents appear to carry the D1327G mutation. Additional sequencing revealed that both subject 4 and subject 5 also carry the two variants. Further analysis showed that the D1327G mutation has already been documented in dbSNP (rs61752484), and it has allele frequency of 0.27% and 0.47% in 1000 Genomes Project and NHLBI-ESP 5400 exomes, respectively. It was not predicted to be deleterious by SIFT (score = 0.4) [59] and PolyPhen (score = 0.07) [60]. Therefore, D1327G does not represent a variant that is private or deleterious to the family.

### Alternative approaches to assess shared candidate variants

We also attempted a different analytical strategy, considering that the analytical procedures described above make strong assumptions on disease mode of inheritance (recessive disease) and the extent of haplotype sharing (IBD = 2 between probands and IBD <2 between probands and unaffected siblings). Instead, prior to implementing the recessive model of inheritance in the last step of Figure 2, we were left with a large collection of rare variants (approximately 500 in each proband) that may be responsible for the autistic phenotype observed in two members of the family. Two hundred of these variants are shared by the two probands, and we directly assessed the likelihood that each variant would be deleterious. Our assumption was that one or several highly penetrant variants in this list could contribute to the pathogenesis of autism in a dominant fashion, and that these variants will not be 100% penetrant so they could still be present in the parents or other unaffected siblings as well.

We submitted this list of variants to wANNOVAR [33], which is a web server that provides a simple and intuitive interface to help users determine the functional significance of variants from high-throughput sequencing data. In addition to allele frequency based filtering to detect rare variants, the wANNOVAR also provides functional prediction scores such as SIFT scores [59], PolyPhen scores [60], PhyloP scores [61], and GERP++ scores [34], to help users determine the functional significance of specific genetic variants. We identified 59 variants that were concordantly predicted as deleterious by SIFT (score <0.05), PolyPhen (score >0.8), and GERP++ (score >2) or without predictions. These include three splicing variants, six frameshift mutations, and 50 non-synonymous variants (Additional file 1: Table S3).

**Figure 3:**
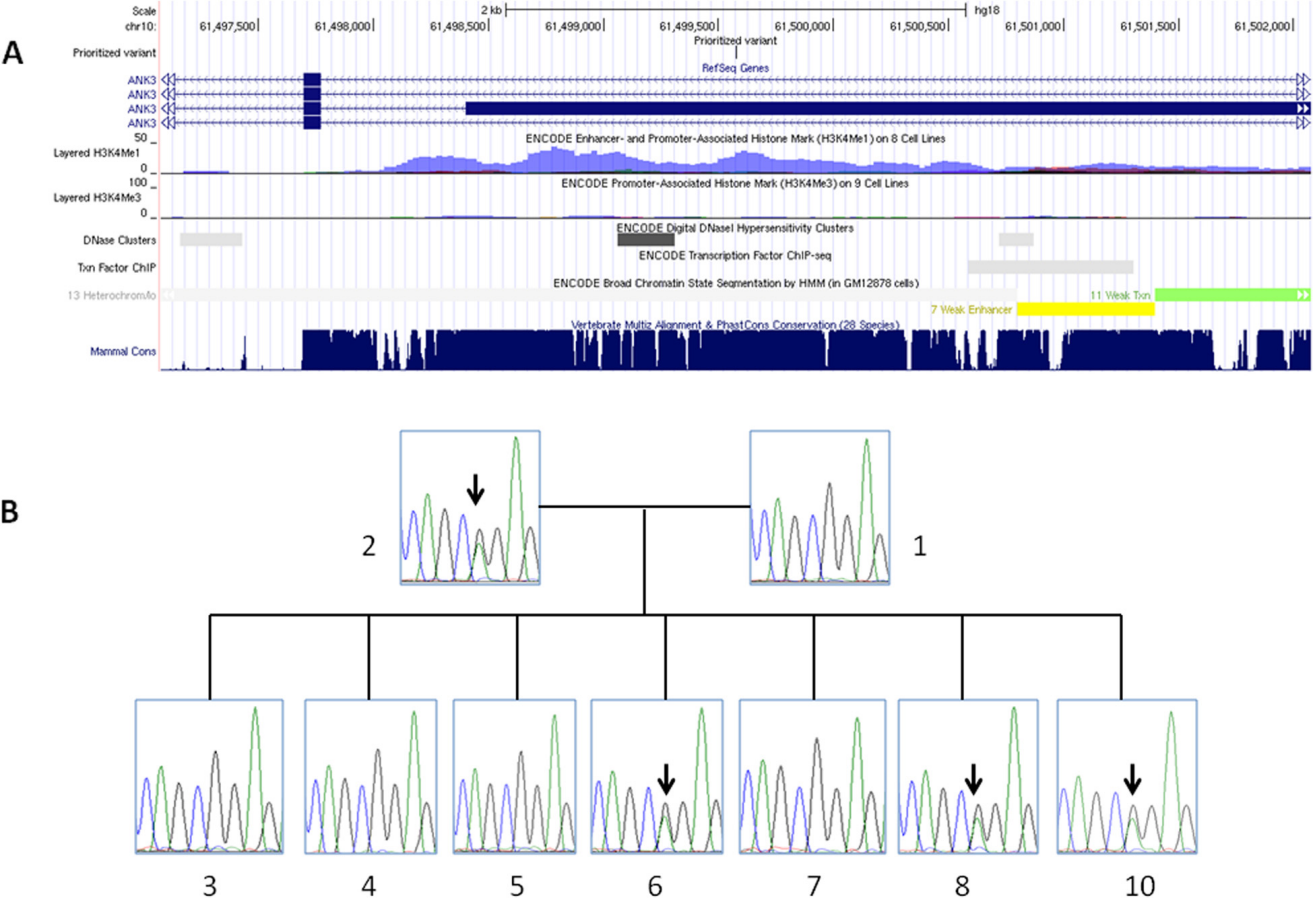
**Illustration of the non-synonymous mutation in ANK3.** (**A**) A UCSC genome browser shot of the *ANK3* gene and the location of the mutation, together with sequence conservation patterns across 28 vertebrate species. (**B**) Validation of the mutation by Sanger sequencing in the family. The primers used are CTTCATGGTCATGGTGGATG (forward) and AGGGGGAAGGGGATAAAAGT (reverse).

We next attempted to further trim down this list of candidate genes, by using prior biological knowledge. Manual examination of the list of genes did not identify any candidate genes that were previously reported in genetic association studies for autism, or were suspected candidate genes for autism. Therefore, we used the DAVID server [62] for functional annotation of these genes, including gene ontology assignment, SwissPro keywords, BioCarta/KEGG pathways, and OMIM association. Among this list of genes, *PTK2B*, *ANK3*, *MYO7A* are involved in neuron differentiation and development based on Gene Ontology. *DCTN1* is associated with neuropathy, amyotrophic lateral sclerosis and Perry syndrome based on OMIM. *MYO7A* is associated with deafness and other neurosensory disorders based on OMIM. Among this prioritized list of genes, the most interesting one is *ANK3* (Ankyrin 3). Several genome-wide association studies for bipolar disorder (BD) have found a strong association of the *ANK3* gene [63,64]. More recently, missense mutations in *ANK3* were identified in four out of 67 patients with ASDs in an exome and candidate gene sequencing study [65], and have been identified in another study that sequenced balanced chromosomal abnormalities in patients with autism or related neurodevelopmental disorders [23]. The ANK3 protein contains two well recognized domains: Ankyrin repeat-containing domain and DEATH domain. The c.11068G > A (p.G3690R) mutation observed in our study is located at the C-terminal end of this large protein, but it does not disrupt either domain. Nevertheless, the variant is located in a large genomic region that is highly conserved across 28 vertebrate species (Figure 3A), suggesting strong evolutionary constraint on the variant. Among the unaffected siblings, only subject 6 shares this variant (Figure 3B).

### Extending to non-coding variants

Our analysis above made the strong assumption that all autism contributory variants in this family might be located in protein coding regions. However, recent large-scale studies such as ENCODE [66] and Roadmap Epigenomics [67] have reinforced an important role for non-coding variants in regulating gene expression and function genome-wide, suggesting that some non-coding variants may also cause diseases with major effects [68]. Compared to previously published autism sequencing studies, one unique aspect of our study is the availability of whole-genome data, so we extended our analysis to non-coding variants. Given that >99% of the variants in whole-genome sequence data are non-coding and that functional prediction algorithms for non-coding variants are far less well developed than coding variants, the data analysis is expected to be much more challenging.

**Figure 4:**
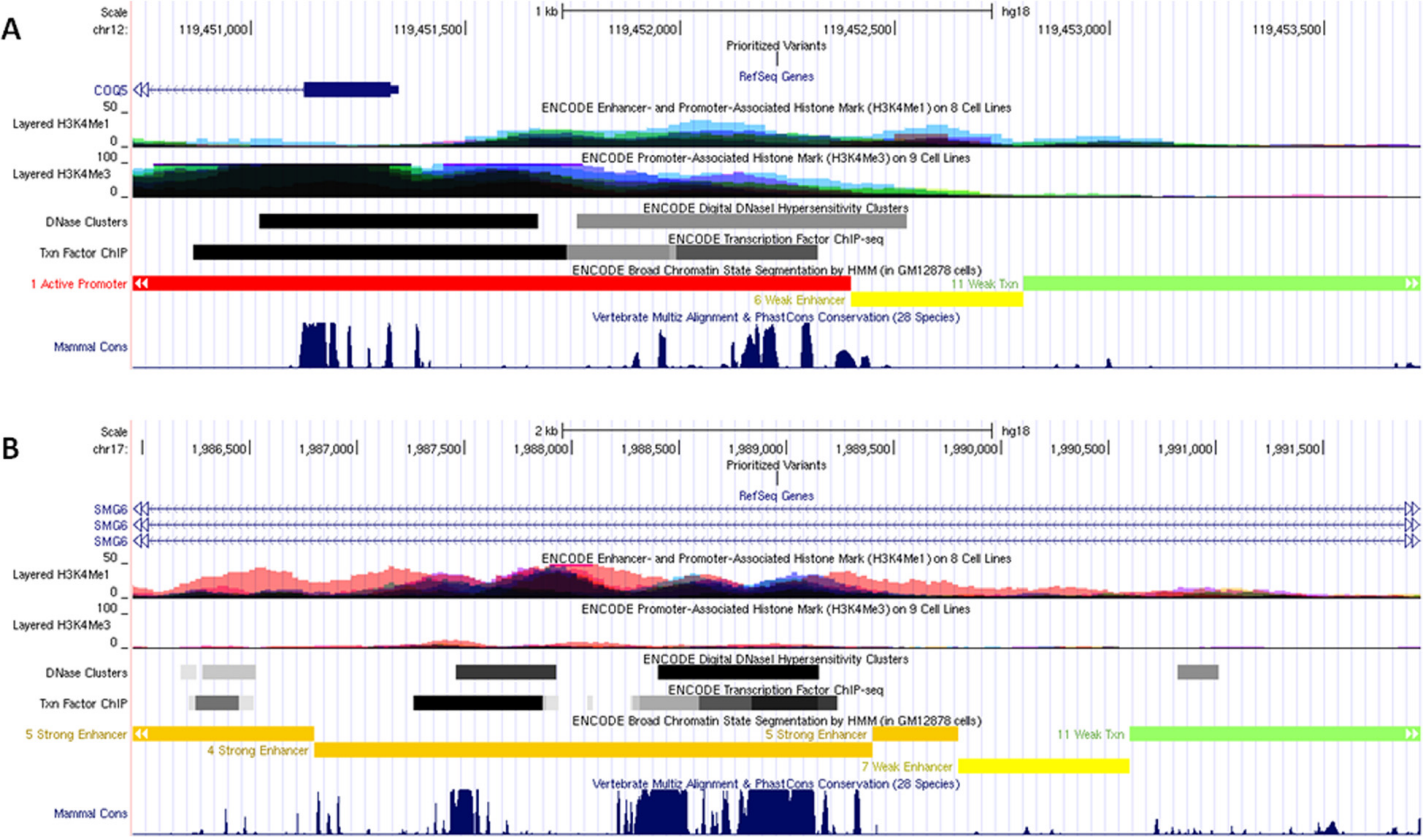
**UCSC genome browser shots of prioritized non-coding variants, demonstrating the sequence conservation levels and the predicted functionality in ENCODE lymphoblastoid cell lines.** The ‘prioritized variants’ track shows the location of the prioritized non-coding variants shared by both probands. (**A**) A prioritized variant is located in a predicted ‘active promoter’ for COQ5. (**B**) A prioritized variant is located in the intronic region of SMG6, and is predicted to be a ‘strong enhancer’.

Nevertheless, we used an analytical procedure aimed to significantly reduce the candidate list and focus on variants that are most likely to be relevant to autism pathogenesis. For each proband, similar to above, we first removed variants that are found in three public databases (1000 Genomes Projects, NHLBI-ESP5400, CG46) with MAF >1%. This resulted in a reduced list of 46,224 non-coding rare variants that are shared between the two probands, which is still more than even the whole-exome variants without any filtering. Next, we attempted to use functional prediction approaches that leverage computational and experimental evidence to prioritize non-coding variants. From the candidate pool, we identified a list of 1,096 variants that are located within GERP++ conserved elements and have GERP++ scores [34] >2, which represent genomic sites that are under strong selective constraint by computational means. Furthermore, we used the ENCODE experimental data to retrieve variants that are located in ‘active promoters’ or ‘strong enhancers’ as predicted by chromHMM [35]. Previous studies demonstrated that disease-associated SNPs are significantly more likely to coincide with the predicted ‘strong enhancers’ [35]. In total, we identified 14 variants located in active promoters and 19 variants located in strong enhancers (Additional file 1: Table S4). Two examples are illustrated (Figure 4). *COQ5* encodes a methyltransferase based on studies in yeast [69]. The intergenic variant upstream of *COQ5* is also highly conserved, and is located within ENCODE H3K4Me1 (enhancer/promoter-associated) and H3K4Me3 (promoter-associated) peaks and DNase I hypersensitivity site (Figure 4A). *SMG6* encodes a protein that participates in the nonsense-mediated mRNA decay (NMD) pathway [70], and it was recently identified as an autism candidate gene by sequencing balanced chromosomal abnormalities in patients with autism or related neurodevelopmental disorders [23]. A prioritized intronic variant is located in a region of *SMG6* that is predicted to be a ‘strong enhancer’, is highly conserved across 28 vertebrate species, and is located in ENCODE H3K4Me1 (enhancer/promoter-associated) peaks and DNase I hypersensitivity sites (Figure 4B).

## Discussion

In this study, we performed a pilot sequencing analysis aimed at identifying potential genetic risk factors for autism in a large pedigree, focusing on inherited mutations. We attempted multiple complementary analytical approaches, each of which identified one to a few candidate genes. We were not able to confirm specific disease-causing mutations with certainty, but we uncovered multiple rare mutations unique to the family, as well as several candidate genes that harbor suspected deleterious coding or non-coding mutations. Among them, based on prior literature, *ANK3* is a highly plausible candidate gene that may increase the susceptibility to ASDs in this family. Given that autism is a complex neuropsychiatric disease, it is likely that multiple contributing variants in the family may increase susceptibility; therefore, even if a specific candidate gene does contribute to disease risk, we caution that a single candidate gene may not be entirely responsible (that is, necessary and sufficient) for the genetic risk of autism in this pedigree. Although our findings are restricted to this specific family, these new candidates can certainly be evaluated in future sequencing studies to establish their true relevance to autism susceptibility.

We applied a whole genome sequencing strategy to reveal specific genetic mutations that may confer susceptibility to ASDs in one single family, and these results can also be compared to exome sequencing studies on schizophrenia, ADHD, and other neurodevelopmental disorders. A recent study revealed that *de-novo* mutation rate might play a major role in schizophrenia, and a large excess of non-synonymous changes were identified by whole exome sequencing from 53 sporadic cases, 22 unaffected controls, and their parents [71]. In another study on schizophrenia, four of the 15 identified *de-novo* mutations in eight probands were nonsense mutations [72]. In a previous small-scale exome sequencing study screening attention deficit/hyperactivity disorder (ADHD) genes on a multiplex pedigree, multiple rare coding variants were identified but were not prioritized based on bioinformatics predictions [42]. In comparison, our study specifically identified rare and family-specific variants rather than *de-novo* mutations.

We initially focused on inherited mutations that are likely to be recessive, which shares some similarity with a very recent exome sequencing study on ASD families enriched for inherited causes due to consanguinity [24]. Other studies have focused on sporadic mutations in families where the parents have been characterized as most likely ‘unaffected’ with autism [17-22], and several observations support the hypothesis that the genetic basis for ASDs in sporadic cases may be different from that seen in families with multiple affected individuals, with some of the former possibly more likely to result from *de-novo* mutation events rather than inherited variants. For an approach complementary to ongoing exome sequencing studies aiming to detect *de-novo* mutations in ASDs [17-22], we specifically selected a multiplex family to test our ability to find inherited mutations that increase risk for ASDs.

In addition to finding inherited mutations, one unique aspect of our study is the use of whole-genome sequence data, which enabled us to perform exploratory analysis on non-coding variants. Given the far larger number of candidate non-coding variants than coding variants, we had to apply highly stringent filtering criteria to focus on those that are most likely to be functionally relevant. These include the use of bioinformatics predictions from evolutionary constraint [34], as well as experimental evidence from the ENCODE project [66]. As our knowledge and bioinformatics approaches for non-coding variants may improve in the future, we may be able to better interrogate the sequencing data to identify disease causal non-coding variants.

We also need to emphasize that previous studies all used the Illumina platform, yet our study used the CG platform, which represents a different type of sequencing technology [28] and generates vastly different types of output files for downstream analysis. As the Illumina platform uses open data formats, a variety of academic and commercial tools have been developed to analyze data from the Illumina sequencers and improve variant calls; in comparison, the CG platform takes a proprietary, ‘black-box’ approach, so that researchers generally have to rely on variant calls and associated quality scores provided by CG. A recent study has comprehensively compared these two platforms and identified that 12% of the called variants are discordant between platforms, yet >60% of these discordant variants were indeed present in the genome based on Sanger validation [40]. Another recently published study also compared data from the 1000 Genomes Project and Complete Genomics, and demonstrated that 19% of the single nucleotide variants (SNVs) reported from common genomes are unique to one dataset [73]. Therefore, current sequencing studies on neuropsychiatric diseases, including ours, may all suffer significantly from false-negative variant calls, and may miss a portion of disease causal variants. Combining data from orthogonal platforms may partially reduce this problem, although this will result in higher sequencing and analytical cost.

In the current study, we first made the assumption that the ASD in the pedigree might be caused by a just a handful of mutations with high penetrance, and under such a model we were able to identify a list of possible such candidate genes. However, in practice, there may be a spectrum of diseases manifesting in each individual, with an as-yet-unknown balance of oligogenic and polygenic modes of inheritance. So, the approaches that we used were somewhat *ad hoc*, and we were unable to generate statistical support for these candidate genes. Indeed, the appropriate statistical threshold to determine functional relevance, in the context of prior biological knowledge, is not well developed. In summary, our study represents one of the first examples demonstrating the feasibility of whole genome sequencing for familial samples and analyzing inherited mutations on ASDs. Ultimately, we believe that studies focusing on *de-novo* or inherited mutations can complement each other, and reveal a more comprehensive picture of susceptibility to ASDs, once sufficient sample sizes have been reached by the community.

## Conclusion

In conclusion, while whole-genome sequencing is a powerful discovery tool, our results demonstrate the complexity of whole-genome analysis when focusing on individual families. Although we were able to generate a list of candidate genes through several approaches, we caution that extensive functional studies are needed to identify any disease causal variants with certainty. Despite that, our analysis provides a prioritized shortlist for further association and validation studies and reflects upon the added value with large family pedigrees.

## Abbreviations

ADHD: Attention deficit hyperactivity disorder; AGRE: Autism Genetic Resource Exchange; ASD: Autism spectrum disorder; CDC: Centers for Disease Control and Prevention; CG: Complete Genomics; CNV: Copy number variation; DEL: Deletion; DUP: Duplication; ENCODE: ENCyclopedia Of DNA Elements; ERDS: Estimation by Read Depth with SNVs; GWAS: Genome-wide association study; IBD: Identity-by-descent; MAF: Minor allele frequency; NCBI: National Center for Biotechnology Information; NGS: Next-generation sequencing; NHLBI: National Heart Lung and Blood Institute; NMD: Nonsense-mediated mRNA decay; PCR: Polymerase chain reaction; SNP: Single nucleotide polymorphism; SNV: Single nucleotide variant; WES: Whole-exome sequencing.

## Competing interests

The authors declare that they have no competing interests.

## Authors’ contributions

LS and KW carried out the data analysis, performed the literature survey, and drafted the manuscript. RG performed alignment and coverage analysis of the whole-genome sequence data, and revised the manuscript. XZ, MH, and GJL interpreted the results and helped with writing of the manuscript. FGO performed the Sanger sequencing validation. CH and CK performed quality control and sample handling. KW and HH conceived the study, guided data analysis, and revised the manuscript. All authors read and approved the final manuscript.

## Supplementary Material

Additional file 1: Table S1 Summary of variant calls generated from whole-genome sequence data on two probands. **Table S2.** A list of highly confident CNV calls generated by ERDS and shared by two probands. Conf refers to ‘confidence score’, and CN refers to ‘copy number’. **Table S3.** A list of prioritized exonic/splicing variants that are shared between two probands and are predicted to be deleterious. **Table S4.** A list of prioritized non-coding variants that are shared between the two probands. Click here for file
