# Supplementary material for: Whole-genome sequencing in an autism multiplex family
Source: Mol Autism. 2013 Apr 18;4:8. doi: 10.1186/2040-2392-4-8 (PMC3642023; doi:10.1186/2040-2392-4-8)
Supplement: Additional file 1: Table S1 — Summary of variant calls generated from whole-genome sequence data on two probands. Table S2. A list of highly confident CNV calls generated by ERDS and shared by two probands. Conf refers to ‘confidence score’, and CN refers to ‘copy number’. Table S3. A list of prioritized exonic/splicing variants that are shared between two probands and are predicted to be deleterious. Table S4. A list of prioritized non-coding variants that are shared between the two probands. [file 2040-2392-4-8-S1.docx]

Table S1. Summary of variant calls generated from whole-genome sequence data on two probands.

| **Identifier** | **10** | **8** |
| --- | --- | --- |
| # variants | 3,811,318 | 3,767,904 |
| # SNPs | 3,396,697 | 3,365,158 |
| Ti/Tv ratio for SNPs | 2.00 | 2.00 |
| Het/Hom ratio for autosome SNPs | 1.46 | 1.45 |
| # exonic variants | 20,278 | 20,710 |
| Ti/Tv ratio for exonic SNPs | 2.89 | 2.82 |
| Het/Hom ratio for exonic autosome SNPs | 1.53 | 1.45 |
| Concordance to SNP arrays | 99.3% | 99.2% |

Table S2. A list of highly confident CNV calls generated by ERDS and shared by two probands. Conf refers to “confidence score”, and CN refers to “copy number”.

| **Region (sample 10)** | **Size** | **Type** | **Conf (sample 10)** | **CN** | **Gene** |
| --- | --- | --- | --- | --- | --- |
| chr4:9820401-9843800 | 23400 | DEL | 2160.94 | 0 | intergenic |
| chr9:23352601-23367800 | 15200 | DEL | 714.14 | 0 | intergenic |
| chr14:40680001-40741000 | 61000 | DEL | 995.8 | 1 | intergenic |
| chr1:72538801-72584800 | 46000 | DEL | 1169.37 | 0 | intergenic |
| chr2:88941601-89014200 | 72600 | DEL | 1614.03 | 1 | intergenic |
| chr3:163990201-164111200 | 121000 | DEL | 4095.74 | 0 | intergenic |
| chr11:48297001-48829000 | 532000 | DUP | 3876.6 | 9 | OR4A47,OR4C3,OR4C45 |
| chr11:49117001-49772000 | 655000 | DUP | 2203.22 | 3 | FOLH1,LOC440040 |
| chr11:50280001-50740000 | 460000 | DUP | 3603.09 | 11 | LOC646813 |
| chr11:51047001-51252000 | 205000 | DUP | 1720.84 | 4 | intergenic |
| chr11:54550001-54797000 | 247000 | DUP | 2104.86 | 15 | TRIM48 |
| chr11:58367001-58643000 | 276000 | DUP | 1491.07 | 3 | FAM111B,GLYATL1,GLYATL2,LOC283194 |
| chr11:88207001-88522000 | 315000 | DUP | 1065.66 | 3 | GRM5 |
| chr21:13516001-14329000 | 813000 | DUP | 5378.93 | 7 | ANKRD20A11P,C21orf15,LOC100288966, MIR3156-3,POTED |
| chr7:4967001-5212000 | 245000 | DUP | 1113.09 | 4 | LOC389458,RBAK,RBAK-LOC389458, RNF216P1,WIPI2,ZNF890P |
| chr7:6816001-7024000 | 208000 | DUP | 1281.74 | 8 | CCZ1B |
| chr7:56626001-56830000 | 204000 | DUP | 966.14 | 3 | intergenic |
| chr7:57159001-57560000 | 401000 | DUP | 2116.73 | 10 | GUSBP10,MIR3147,ZNF479,ZNF716 |
| chr7:61605001-62212000 | 607000 | DUP | 3723.16 | 40 | intergenic |
| chr7:63095001-63489000 | 394000 | DUP | 1637.49 | 3 | LOC100506050,ZNF679,ZNF727,ZNF735,ZNF736 |
| chr7:65059001-65307000 | 248000 | DUP | 986.78 | 3 | ASL,CRCP,GUSB |
| chr7:65746001-66023000 | 277000 | DUP | 1428.65 | 3 | GTF2IRD1P1,RABGEF1 |
| chr7:66062001-66520000 | 458000 | DUP | 2863.13 | 4 | MIR4650-1,MIR4650-2,PMS2P4,SBDS,STAG3L4,TYW1 |
| chr7:71633001-71957000 | 324000 | DUP | 1972.53 | 3 | MIR4650-1,MIR4650-2,SBDSP1,TYW1B |
| chr7:75107001-75415000 | 308000 | DUP | 1191.58 | 3 | CCL24,CCL26,HIP1,MIR4651,POR,RHBDD2,SNORA14A |
| chr7:97291001-97545000 | 254000 | DUP | 1214.73 | 7 | ASNS,MGC72080,OCM2 |
| chr7:98580001-98837000 | 257000 | DUP | 1000.81 | 3 | ARPC1A,ARPC1B,KPNA7,MYH16,PDAP1 |
| chr7:127884001-128087000 | 203000 | DUP | 1412.5 | 6 | FLJ45340,HILPDA,METTL2B |
| chr17:20397001-20616000 | 219000 | DUP | 1702.12 | 4 | CDRT15L2,LOC100287072 |
| chr17:25982001-26183000 | 201000 | DUP | 1381.99 | 4 | CRLF3,LRRC37BP1,SUZ12P1 |
| chr2:132275001-132485000 | 210000 | DUP | 1228.62 | 6 | C2orf27B |
| chr22:15257001-15462000 | 205000 | DUP | 937.83 | 10 | CCT8L2 |
| chr1:83375001-83627000 | 252000 | DUP | 752.39 | 3 | intergenic |
| chr1:119740001-119963000 | 223000 | DUP | 1181.63 | 3 | HSD3B1,HSD3B2,HSD3BP4,LINC00622 |
| chr16:28988001-29277000 | 289000 | DUP | 2464.46 | 4 | RRN3P2,SNX29P2 |
| chr16:32247001-32566000 | 319000 | DUP | 2851.26 | 8 | intergenic |
| chr16:69403001-69760000 | 357000 | DUP | 4597.6 | 4 | HYDIN,HYDIN2 |
| chr13:40179001-40391000 | 212000 | DUP | 889.79 | 3 | MIR320D1,MIR621,MRPS31,SLC25A15, SUGT1P3,TPTE2P50 |
| chr6:57398001-57717000 | 319000 | DUP | 2642.9 | 4 | PRIM2 |
| chr6:57794001-58053000 | 259000 | DUP | 918.11 | 3 | intergenic |
| chr6:58266001-58498000 | 232000 | DUP | 1274.05 | 5 | GUSBP4 |
| chr6:58509001-58883000 | 374000 | DUP | 1271.84 | 5 | intergenic |
| chr12:8205001-8455000 | 250000 | DUP | 1221.39 | 12 | FAM66C,FAM86FP,FAM90A1,LOC389634,ZNF705A |
| chr12:34403001-34640000 | 237000 | DUP | 1119.05 | 4 | intergenic |
| chr12:36143001-36676000 | 533000 | DUP | 2295 | 15 | intergenic |
| chr12:62210001-62433000 | 223000 | DUP | 1256.11 | 4 | DPY19L2 |
| chr15:18652001-18888000 | 236000 | DUP | 1765.97 | 7 | CHEK2P2,HERC2P3 |
| chr15:21884001-22375000 | 491000 | DUP | 3262.45 | 4 | PWRN1,PWRN2 |
| chr4:70073001-70354000 | 281000 | DUP | 1261.56 | 4 | UGT2B11,UGT2B28 |
| chr4:132903001-133119000 | 216000 | DUP | 593.41 | 8 | intergenic |
| chr4:145028001-145241000 | 213000 | DUP | 1089.16 | 4 | GYPB,GYPE |
| chr10:30793001-31071000 | 278000 | DUP | 916.65 | 3 | LYZL2 |
| chr10:38493001-38812000 | 319000 | DUP | 1845.78 | 6 | HSD17B7P2,LOC100129055,LOC399744,SEPT7L |
| chr10:46367001-46577000 | 210000 | DUP | 2675.47 | 4 | GPRIN2,HNRNPA1P33,LINC00842,PPYR1,SYT15 |
| chr19:19746001-19972000 | 226000 | DUP | 1686.26 | 4 | LINC00663,ZNF253,ZNF506,ZNF93 |
| chr19:19993001-20598000 | 605000 | DUP | 4975.33 | 3 | MIR1270-1,MIR1270-2,ZNF486,ZNF626, ZNF682,ZNF737,ZNF826P,ZNF90 |
| chr19:24130001-24395000 | 265000 | DUP | 1775.21 | 6 | HAVCR1P1 |
| chr19:32424001-32827000 | 403000 | DUP | 3025.27 | 61 | intergenic |
| chr19:57669001-58005000 | 336000 | DUP | 3161.23 | 4 | ZNF137P,ZNF28,ZNF578,ZNF600,ZNF611, ZNF701,ZNF808,ZNF83 |
| chr19:58747001-59004000 | 257000 | DUP | 1196.68 | 3 | 54 genes |
| chr5:46154001-46441000 | 287000 | DUP | 1381.5 | 17 | intergenic |

Table S3. A list of prioritized exonic/splicing variants that are shared between two probands and are predicted to be deleterious.

| **Type** | **Gene** | **Function** | **Amino Acid Change** | **Chr** | **Start** | **End** | **Ref** | **Obs** |
| --- | --- | --- | --- | --- | --- | --- | --- | --- |
| exonic | NOTCH2 | nonsynonymous SNV | NM_024408:c.C6139T:p.R2047W | 1 | 120,260,729 | 120,260,729 | G | A |
| exonic | DCTN1 | nonsynonymous SNV | NM_001135041:c.G473A:p.R158H | 2 | 74,451,429 | 74,451,429 | C | T |
| exonic | CWC22 | nonsynonymous SNV | NM_020943:c.G37T:p.G13C | 2 | 180,561,607 | 180,561,607 | C | A |
| exonic | PLCD4 | nonsynonymous SNV | NM_032726:c.G1984A:p.V662I | 2 | 219,208,850 | 219,208,850 | G | A |
| exonic | CCR5 | nonsynonymous SNV | NM_000579:c.G316A:p.G106R | 3 | 46,389,713 | 46,389,713 | G | A |
| exonic | TKT | nonsynonymous SNV | NM_001064:c.A1306G:p.M436V | 3 | 53,238,152 | 53,238,152 | T | C |
| exonic | ZNF717 | frameshift substitution | ZNF717:NM_001128223:exon5:c.622_622delinsGC | 3 | 75,870,842 | 75,870,842 | T | GC |
| exonic | CEP97 | nonsynonymous SNV | NM_024548:c.T1198C:p.S400P | 3 | 102,959,338 | 102,959,338 | T | C |
| exonic | P2RY13 | nonsynonymous SNV | NM_176894:c.G35A:p.S12N | 3 | 152,529,972 | 152,529,972 | C | T |
| exonic | MUC4 | nonsynonymous SNV | NM_018406:c.C7601A:p.A2534D | 3 | 196,995,245 | 196,995,245 | G | T |
| exonic | NFXL1 | nonsynonymous SNV | NM_152995:c.G7A:p.A3T | 4 | 47,610,971 | 47,610,971 | C | T |
| exonic | INTS12 | nonsynonymous SNV | NM_001142471:c.A107G:p.D36G | 4 | 106,840,505 | 106,840,505 | T | C |
| exonic | LRIT3 | nonsynonymous SNV | NM_198506:c.A452G:p.Y151C | 4 | 110,992,444 | 110,992,444 | A | G |
| exonic | KIAA0947 | nonsynonymous SNV | NM_015325:c.A723T:p.R241S | 5 | 5,510,476 | 5,510,476 | A | T |
| exonic | NIPBL | nonsynonymous SNV | NM_015384:c.A2450T:p.D817V | 5 | 37,021,489 | 37,021,489 | A | T |
| exonic | NLN | nonsynonymous SNV | NM_020726:c.C1450T:p.P484S | 5 | 65,124,161 | 65,124,161 | C | T |
| exonic | ERAP1 | frameshift deletion | NM_001040458:c.1595delT:p.L532fs | 5 | 96,150,074 | 96,150,074 | A | - |
| exonic | P4HA2 | nonsynonymous SNV | NM_001017973:c.C691T:p.R231C | 5 | 131,573,894 | 131,573,894 | G | A |
| exonic | PCDHGA11 | nonsynonymous SNV | NM_018914:c.T289G:p.C97G | 5 | 140,781,267 | 140,781,267 | T | G |
| exonic | HAVCR2 | nonsynonymous SNV | NM_032782:c.A245G:p.Y82C | 5 | 156,466,365 | 156,466,365 | T | C |
| exonic | EFHC1 | nonsynonymous SNV | NM_018100:c.G458A:p.R153Q | 6 | 52,411,233 | 52,411,233 | G | A |
| exonic | DOPEY1 | nonsynonymous SNV | NM_015018:c.G7241A:p.R2414Q | 6 | 83,934,448 | 83,934,448 | G | A |
| exonic | RFX6 | nonsynonymous SNV | NM_173560:c.C2261T:p.P754L | 6 | 117,355,258 | 117,355,258 | C | T |
| exonic | DNAH11 | nonsynonymous SNV | NM_003777:c.G11419C:p.D3807H | 7 | 21,870,702 | 21,870,702 | G | C |
| exonic | ZNF727 | nonsynonymous SNV | NM_001159522:c.A1260T:p.E420D | 7 | 63,176,122 | 63,176,122 | A | T |
| exonic | CPA2 | nonsynonymous SNV | NM_001869:c.C911T:p.T304I | 7 | 129,706,662 | 129,706,662 | C | T |
| exonic | PSD3 | nonsynonymous SNV | NM_206909:c.C469A:p.L157I | 8 | 18,701,096 | 18,701,096 | G | T |
| exonic | PTK2B | nonsynonymous SNV | NM_173175:c.T1051C:p.Y351H | 8 | 27,346,932 | 27,346,932 | T | C |
| exonic | HAUS6 | nonsynonymous SNV | NM_017645:c.T1202A:p.L401H | 9 | 19,066,692 | 19,066,692 | A | T |
| exonic | FAM75D1 | nonsynonymous SNV | NM_001001670:c.T1742A:p.L581Q | 9 | 83,796,947 | 83,796,947 | T | A |
| exonic | DPP7 | nonsynonymous SNV | NM_013379:c.C809T:p.A270V | 9 | 139,127,287 | 139,127,287 | G | A |
| exonic | WDFY4 | nonsynonymous SNV | NM_020945:c.C6497T:p.T2166M | 10 | 49,708,907 | 49,708,907 | C | T |
| exonic | ANK3 | nonsynonymous SNV | NM_020987:c.G11068A:p.G3690R | 10 | 61,499,577 | 61,499,577 | C | T |
| exonic | LIPK | nonsynonymous SNV | NM_001080518:c.T370A:p.S124T | 10 | 90,480,866 | 90,480,866 | T | A |
| exonic | MUC6 | frameshift substitution | MUC6:NM_005961:exon31:c.4706_4706delinsTA | 11 | 1,008,095 | 1,008,095 | G | TA |
| exonic | MYO7A | nonsynonymous SNV | NM_000260:c.C4450A:p.L1484I | 11 | 76,587,196 | 76,587,196 | C | A |
| exonic | MAML2 | nonsynonymous SNV | NM_032427:c.A2222G:p.Y741C | 11 | 95,364,453 | 95,364,453 | T | C |
| exonic | CACNA2D4 | nonsynonymous SNV | NM_172364:c.G2008A:p.G670S | 12 | 1,838,004 | 1,838,004 | C | T |
| exonic | KNTC1 | nonsynonymous SNV | NM_014708:c.A3115G:p.I1039V | 12 | 121,633,337 | 121,633,337 | A | G |
| exonic | ABHD12B | nonsynonymous SNV | NM_001206673:c.T128C:p.M43T | 14 | 50,414,443 | 50,414,443 | T | C |
| exonic | SPTB | nonsynonymous SNV | NM_000347:c.T5726C:p.F1909S | 14 | 64,307,428 | 64,307,428 | A | G |
| exonic | ZC2HC1C | frameshift insertion | NM_001042430:c.46_47insA:p.M16fs | 14 | 74,607,075 | 74,607,075 | - | A |
| exonic | AHNAK2 | nonsynonymous SNV | NM_138420:c.C15140T:p.P5047L | 14 | 104,477,693 | 104,477,693 | G | A |
| exonic | BAHD1 | nonsynonymous SNV | NM_014952:c.T929A:p.L310Q | 15 | 38,538,884 | 38,538,884 | T | A |
| exonic | THSD4 | nonsynonymous SNV | NM_024817:c.C103T:p.P35S | 15 | 69,294,431 | 69,294,431 | C | T |
| exonic | GEMIN4 | nonsynonymous SNV | NM_015721:c.G974A:p.R325Q | 17 | 597,059 | 597,059 | C | T |
| exonic | PROCA1 | frameshift insertion | NM_152465:c.956_957insC:p.A319fs | 17 | 24,054,758 | 24,054,758 | - | G |
| exonic | KRTAP16-1 | nonsynonymous SNV | NM_001146182:c.C101T:p.S34F | 17 | 36,718,931 | 36,718,931 | G | A |
| exonic | COASY | nonsynonymous SNV | NM_025233:c.G156C:p.Q52H | 17 | 37,968,322 | 37,968,322 | G | C |
| exonic | DNAH17 | nonsynonymous SNV | NM_173628:c.C9776T:p.A3259V | 17 | 73,966,763 | 73,966,763 | G | A |
| exonic | ABCA7 | nonsynonymous SNV | NM_019112:c.G5963T:p.C1988F | 19 | 1,015,171 | 1,015,171 | G | T |
| exonic | PLIN4 | nonsynonymous SNV | NM_001080400:c.C3943T:p.R1315W | 19 | 4,455,602 | 4,455,602 | G | A |
| exonic | RASAL3 | nonsynonymous SNV | NM_022904:c.C1877T:p.A626V | 19 | 15,426,549 | 15,426,549 | G | A |
| exonic | SIGLEC12 | frameshift substitution | SIGLEC12:NM_053003:exon1:c.193_193delinsAG | 19 | 56,696,607 | 56,696,607 | G | CT |
| exonic | ZNF534 | nonsynonymous SNV | NM_001143938:c.G271A:p.E91K | 19 | 57,630,274 | 57,630,274 | G | A |
| exonic | ASMT | nonsynonymous SNV | NM_001171038:c.A44C:p.Y15S | X | 1,694,136 | 1,694,136 | A | C |
| splicing | CNBD1 |  |  | 8 | 88,287,484 | 88,287,484 | T | C |
| splicing | GBA2 |  |  | 9 | 35,731,063 | 35,731,063 | T | C |
| splicing | EMG1 |  |  | 12 | 6,950,473 | 6,950,473 | T | GC |

Table S4. A list of prioritized non-coding variants that are shared between the two probands.

| Function type (ENCODE chromHMM) | Gene (within gene or distance to nearby genes) | Chr | Start | End | Ref Allele | Observed Allele | Type | Variant quality (Sample 8) |
| --- | --- | --- | --- | --- | --- | --- | --- | --- |
| Active promoter | SYT11 | 1 | 154,096,491 | 154,096,491 | G | C | snp | 62 |
| Active promoter | DTL | 1 | 210,275,738 | 210,275,738 | T | C | snp | 171 |
| Active promoter | E2F3 | 6 | 20,512,179 | 20,512,179 | G | A | snp | 121 |
| Active promoter | HIST1H4H (dist=42157) BTN3A2 (dist=37514) | 6 | 26,435,863 | 26,435,863 | A | G | snp | 112 |
| Active promoter | TRERF1 | 6 | 42,527,688 | 42,527,688 | C | T | snp | 110 |
| Active promoter | MARCKS | 6 | 114,285,498 | 114,285,498 | T | A | snp | 48 |
| Active promoter | VCP;FANCG | 9 | 35,062,936 | 35,062,936 | G | T | snp | 410 |
| Active promoter | FAM118B | 11 | 125,586,910 | 125,586,910 | G | T | snp | 249 |
| Active promoter | KCNA1 | 12 | 4,890,338 | 4,890,340 | GAG | - | del | 267 |
| Active promoter | COQ5 | 12 | 119,452,227 | 119,452,227 | C | A | snp | 195 |
| Active promoter | HIF1A | 14 | 61,232,515 | 61,232,515 | G | A | snp | 161 |
| Active promoter | SMG6 | 17 | 2,088,815 | 2,088,815 | T | C | snp | 305 |
| Active promoter | MEOX1 | 17 | 39,094,580 | 39,094,580 | A | - | del | 68 |
| Active promoter | ACTG1 | 17 | 77,093,364 | 77,093,364 | G | C | snp | 384 |
| Strong enhancer | DUSP10 | 1 | 219,966,477 | 219,966,477 | G | A | snp | 120 |
| Strong enhancer | FAM43A (dist=2180) LOC100507391 (dist=17204) | 3 | 195,893,235 | 195,893,235 | C | A | snp | 351 |
| Strong enhancer | ZNF608 (dist=667738) GRAMD3 (dist=947245) | 5 | 124,776,442 | 124,776,442 | T | C | snp | 241 |
| Strong enhancer | FSTL4 (dist=213418) C5orf15 (dist=129557) | 5 | 133,189,540 | 133,189,540 | C | G | snp | 279 |
| Strong enhancer | CYFIP2 | 5 | 156,628,191 | 156,628,191 | G | A | snp | 177 |
| Strong enhancer | CYP7B1 (dist=118008) LINC00251 (dist=244024) | 8 | 65,991,910 | 65,991,910 | G | A | snp | 326 |
| Strong enhancer | MOB3B | 9 | 27,356,276 | 27,356,276 | A | G | snp | 266 |
| Strong enhancer | ARID5B | 10 | 63,449,200 | 63,449,200 | T | C | snp | 188 |
| Strong enhancer | KNTC1 | 12 | 121,597,117 | 121,597,117 | T | C | snp | 282 |
| Strong enhancer | STARD13 (dist=51577) RFC3 (dist=89697) | 13 | 33,200,509 | 33,200,509 | A | C | snp | 336 |
| Strong enhancer | TNFSF13B | 13 | 107,720,199 | 107,720,199 | G | T | snp | 165 |
| Strong enhancer | RAD51B | 14 | 67,718,581 | 67,718,581 | C | T | snp | 305 |
| Strong enhancer | SEC11A (dist=8596) ZNF592 (dist=23548) | 15 | 83,069,274 | 83,069,274 | G | A | snp | 197 |
| Strong enhancer | LRRC28 (dist=126234) MEF2A (dist=53399) | 15 | 97,870,255 | 97,870,257 | GCC | CCT | sub | 346 |
| Strong enhancer | CBFA2T3 | 16 | 87,515,116 | 87,515,116 | A | G | snp | 441 |
| Strong enhancer | SMG6 | 17 | 1,988,955 | 1,988,955 | G | A | snp | 130 |
| Strong enhancer | TP53INP2 | 20 | 32,763,274 | 32,763,274 | C | T | snp | 529 |
| Strong enhancer | NCOA3 | 20 | 45,568,596 | 45,568,596 | G | A | snp | 121.5 |
| Strong enhancer | MED15 | 22 | 19,235,575 | 19,235,575 | G | A | snp | 215 |
